# Preventing substance misuse: study protocol for a randomised controlled trial of the Strengthening Families Programme 10–14 UK (SFP 10–14 UK)

**DOI:** 10.1186/1471-2458-14-49

**Published:** 2014-01-17

**Authors:** Jeremy Segrott, David Gillespie, Jo Holliday, Ioan Humphreys, Simon Murphy, Ceri Phillips, Hayley Reed, Heather Rothwell, David Foxcroft, Kerenza Hood, Zoe Roberts, Jonathan Scourfield, Claire Thomas, Laurence Moore

**Affiliations:** 1 Centre for the Development and Evaluation of Complex Interventions for Public Health Improvement (DECIPHer), Cardiff School of Social Sciences, Cardiff University, 1-3 Museum Place, CF10 3BD Cardiff, UK; 2 South East Wales Trials Unit, School of Medicine, Cardiff University, Neuadd Meirionnydd, Heath Park, CF14 4YS Cardiff, UK; 3 Swansea Centre for Health Economics; College of Health and Human Sciences, Swansea University, Singleton Park, SA2 8PP Swansea, UK; 4 Faculty of Health and Life Sciences, Oxford Brookes University, Jack Straw’s Lane, OX3 0FL Oxford, UK; 5 Institute of Primary Care and Public Health, Cardiff University, Neuadd Meirionnydd, Heath Park, CF14 4YS Cardiff, UK; 6 Cardiff School of Social Sciences, Cardiff University, Glamorgan Building, King Edward VII Avenue, CF10 3WT Cardiff, UK; 7 MRC/CSO Social and Public Health Sciences Unit, University of Glasgow, 4 Lilybank Gardens, G12 8RZ Glasgow, UK

**Keywords:** Substance misuse, Alcohol, Prevention, Strengthening Families Programme 10–14, SFP 10–14 UK, Young people

## Abstract

**Background:**

Prevention of alcohol, drug and tobacco misuse by young people is a key public health priority. There is a need to develop the evidence base through rigorous evaluations of innovative approaches to substance misuse prevention. The Strengthening Families Programme 10–14 is a universal family-based alcohol, drugs and tobacco prevention programme, which has achieved promising results in US trials, and which now requires cross-cultural assessment. This paper therefore describes the protocol for a randomised controlled trial of the UK version of the Strengthening Families Programme 10–14 (SFP 10–14 UK).

**Methods/Design:**

The trial comprises a pragmatic cluster randomised controlled effectiveness trial with families as the unit of randomisation, with embedded process and economic evaluations. Participating families will be randomised to one of two treatment groups - usual care with full access to existing services (control group), or usual care plus SFP 10–14 UK (intervention group). The trial has two primary outcomes - the number of occasions that young people report having drunk alcohol in the last 30 days, and drunkenness during the last 30 days, both dichotomised as ‘never’ and ‘1-2 times or more’. The main follow-up is at 2 years past baseline, and short-term and intermediate outcomes are also measured at 9 and 15 months.

**Discussion:**

The results from this trial will provide evidence on the effectiveness and cost-effectiveness of an innovative universal family-based substance misuse prevention programme in a UK context.

**Trial registration:**

Current Controlled Trials ISRCTN63550893.

## Background

Risk behaviour by young people, including substance misuse (alcohol, tobacco and illegal drugs), antisocial behaviour and crime has a substantial impact on the UK economy and the health of its population. These behaviours are associated with morbidity and mortality among young people, poor education, social exclusion, teenage pregnancy, conduct disorders and poor health over the life course [1-3]. The 2009/10 HBSC survey in Wales indicated that at age 15, 50% of girls and 47% of boys reported that they had been drunk twice or more (the third highest of 38 countries); 16% of girls and 11% of boys reported that they smoked cigarettes at least once a week; and 20% of girls and 22% of boys reported that they had ever used cannabis. Concerning drunkenness and cannabis use prevalence, Wales, Scotland and England all had rates above the HBSC average [4].

Intervention efforts have largely focussed on individual risk behaviours, ignoring the co-occurrence of multiple health risk behaviours [5]. In the 2012 survey of drug use, smoking and drinking among 11–15 year olds in England, 6% of respondents had smoked during the last week (rising to 15% by age 15) [6]. Past week smoking was strongly associated with other risk behaviours, and “[o]f the 6% of pupils who reported smoking in the last week, most (5% of all pupils) had also drunk alcohol or taken drugs recently, or had done both” (p185).

The survey also found that truanting from school was linked to these behaviours. For instance, “[p]upils who had truanted from school were more likely to be regular smokers compared with pupils who had never truanted (odds ratio = 2.02)” (p36).

Understanding the co-occurrence of multiple health risk behaviours and their association with poor educational outcomes, social disadvantage, conduct disorders and poor physical and emotional health is therefore an important area of current public health research [5,7-10]. Similarly, there is increased interest in prevention efforts which focus on risk and protective factors such as pro-social behaviour, resilience and positive youth development [11-13], and which therefore have the potential to impact upon multiple risk outcomes. Family-based risk and protective factors have been identified as important targets for intervention [14], including the modelling of parents’ alcohol use [15], rules and monitoring around substance use [16,17], and more general parenting styles and relationships within families [18-20]. Parenting and family interventions form a central part of many governments’ health, welfare and education policies, and prevention of substance misuse is also a key priority [21-25]. Yet interventions are often developed without reference to the existing evidence base or theoretical frameworks, and implemented without rigorous evaluation [26].

A promising programme which has targeted multiple risk behaviours by addressing risk and protective factors located in the family is the Strengthening Families Programme 10–14 (SFP 10–14). It draws on theories of bio-psychosocial vulnerability, resiliency and family process, and was initially developed as the 14-session Strengthening Families Programme for high-risk families with children aged 6–12 years whose parents were misusing alcohol or drugs. The revised SFP 10–14 is a seven week universal programme targeting young people aged 10 to 14 years and their parents [27]. SFP 10–14 has been reported to be effective over a six-year follow-up period in promoting family integration, delaying the onset of alcohol use, reducing uptake of smoking, the incidence of harder drug use (methamphetamine) [28-31] and substance use at 10 year follow-up (aged 21) [32]. Delaying the age of initiation of substance use is an important goal, since epidemiological research has indicated that later onset of alcohol and drug use is associated with reduced lifetime prevalence of alcohol and drug problems [33-35].

US-based cost-benefit assessments indicate that SFP 10–14 can be cost-effective at the population level for preventing alcohol misuse, other alcohol problems and also for tobacco use [36-38], cannabis and other drug use prevention. A potential ten-fold return for every dollar invested in the programme has been reported [36], although the authors conclude that economic analysis had largely been unexplored in the evaluation of such interventions. Another US study that examined the SFP 10–14 alongside other school-based programmes has shown that the costs of participation amounted to US$150 while the benefits realised could be as much as US$1000 per participant, depending on the extent of drug use [38]. The emphasis in these studies has been on the cost-benefit ratio and whether prevention can generate returns on investment from avoidance of other costs in other sectors and in future years. The results of such studies are highly dependent on the perspective taken and the nature and range of assumptions made. Most critically, they are dependent on the existence of high quality investigations that utilise reliable and valid outcome measures to produce indications of effect and that yield detailed and relevant cost information from a range of perspectives. Well conducted studies of this nature can contribute to subsequent meta-analyses and modelling exercises to identify efficient policy options.

The current evidence base for SFP 10–14 is derived exclusively from the US from two trials conducted by the same research team. Recent Cochrane systematic reviews and a 2006 technical report to the *World Health Organisation (WHO) Expert Committee on Problems Related to Alcohol Consumption* recommend that the SFP 10–14 is adapted and tested in other cultures and settings before widespread implementation [39,40]. Recent methodological critiques of the two existing RCTs of SFP 10–14 point to limitations in the statistical analyses by the original research team, namely the use of multiple and one sided statistical tests leading to claims of statistical significance at p < 0.05 that may not be justified [41-45]. In these critiques the key issue is statistical precision, and an intention-to-treat re-analysis of the original data for a Cochrane review highlighted that the results of the SFP 10–14 trials showed an important effect with low precision [39]. For example, in this re-analysis the Absolute Risk Reduction and 95% confidence interval for drunkenness was 11.27% (0.31% to 22.24%). This underlines the need for additional, high quality trials to assess effectiveness and cost-effectiveness with precision and determine whether the programme has long-term public health benefits. This view is reinforced by a recent independent UK Department of Health-commissioned review of prevention and behaviour change in young people, which concluded that “More RCTs [are needed] of the Strengthening Families Program” [46].

It is not clear whether the apparent effectiveness of the SFP 10–14 in reducing alcohol and drug problems at the population level, as shown in research from the United States, will translate to other cultures, settings and countries. The SFP 10–14 has recently been culturally adapted for use in the UK [47,48] and there is substantial UK policy interest in the programme. This includes the use of the SFP 10–14 as part of the UK Government’s Family Intervention Projects across a small number of selected sites in England, which target high risk families [49]. An evaluation of the programme’s implementation in Barnsley, England (which mainly used the original US programme materials), highlighted the need for some cultural adaptation (now completed) and consideration of the best approach with regards to universal or high-risk targeting [50,51]. Many studies have highlighted the challenges associated with targeted programmes for young people, including recruitment, stigmatisation, and managing group dynamics, with the potential for harmful peer modelling effects [52-58]. Such targeting also risks losing the beneficial group effects which are achieved through the universal delivery of programmes such as SFP 10–14 to whole school populations, as in the US SFP10-14. In Cardiff, Wales, UK, these problems led to the development of an innovative approach of providing the SFP 10–14 as a universal intervention (available to any family via practitioner referral or family application), and running mixed groups of families from the ‘general population’ in combination with families with challenges (in the context of a group setting) [59], with the aim of promoting positive group dynamics. Groups of families attending the SFP therefore normally comprise around 70% from the general population, and 30% who may experience or present challenges in a group setting (families with challenges). It should be noted that in this ‘mixed families’ approach, such challenges relate specifically to the delivery and receipt of the group sessions, rather than general characteristics of participants’ support needs, family functioning or other risk factors. Examples of the challenges experienced or presented by families therefore include issues such as low literacy levels, behavioural problems (e.g. anger/aggression) and exclusion of children from school (and who may find working in a group setting challenging). An earlier paper describes the mixed families approach in more detail [59]. These mixed groups demonstrated evidence of high levels of participant retention and acceptability, and social effects outside the programme, with participating families supporting each other between sessions and after the end of the programme. Our trial of the SFP 10–14 UK in Wales (Project SFP Cymru) uses this ‘mixed families’ approach to group recruitment and composition, as well as the fully culturally adapted materials. The trial therefore builds on the completed formative evaluation, adaptation and exploratory evaluation of the UK version of the programme [47,48,50,51,59], and will generate important new evidence on the programme’s long-term effectiveness and cost-effectiveness in the UK.

## Methods/design

### Study design

The trial comprises a pragmatic cluster randomised controlled effectiveness trial with families as the unit of randomisation, with embedded process and economic evaluations.

### Recruitment

SFP 10–14 UK will be implemented in seven geographical areas of Wales by local agencies that are independent of the research team (e.g. local authority parenting teams, children’s charities). Trial areas will run the programme on approximately 8 occasions over a two year period, with a maximum capacity of 10–12 intervention families per programme.

SFP 10–14 UK is designed for delivery to families with children aged 10–14, and in the Wales adaptation is targeted towards a mix of children and their families. Each programme is open to families from a loosely defined and fairly large geographical area. In each area, self-referrals to the Programme come forward in response to awareness-raising in community and educational settings. Other referrals come from agencies such as education, health and social services which identify families that may benefit from participating in the Programme.

**Figure 1:**
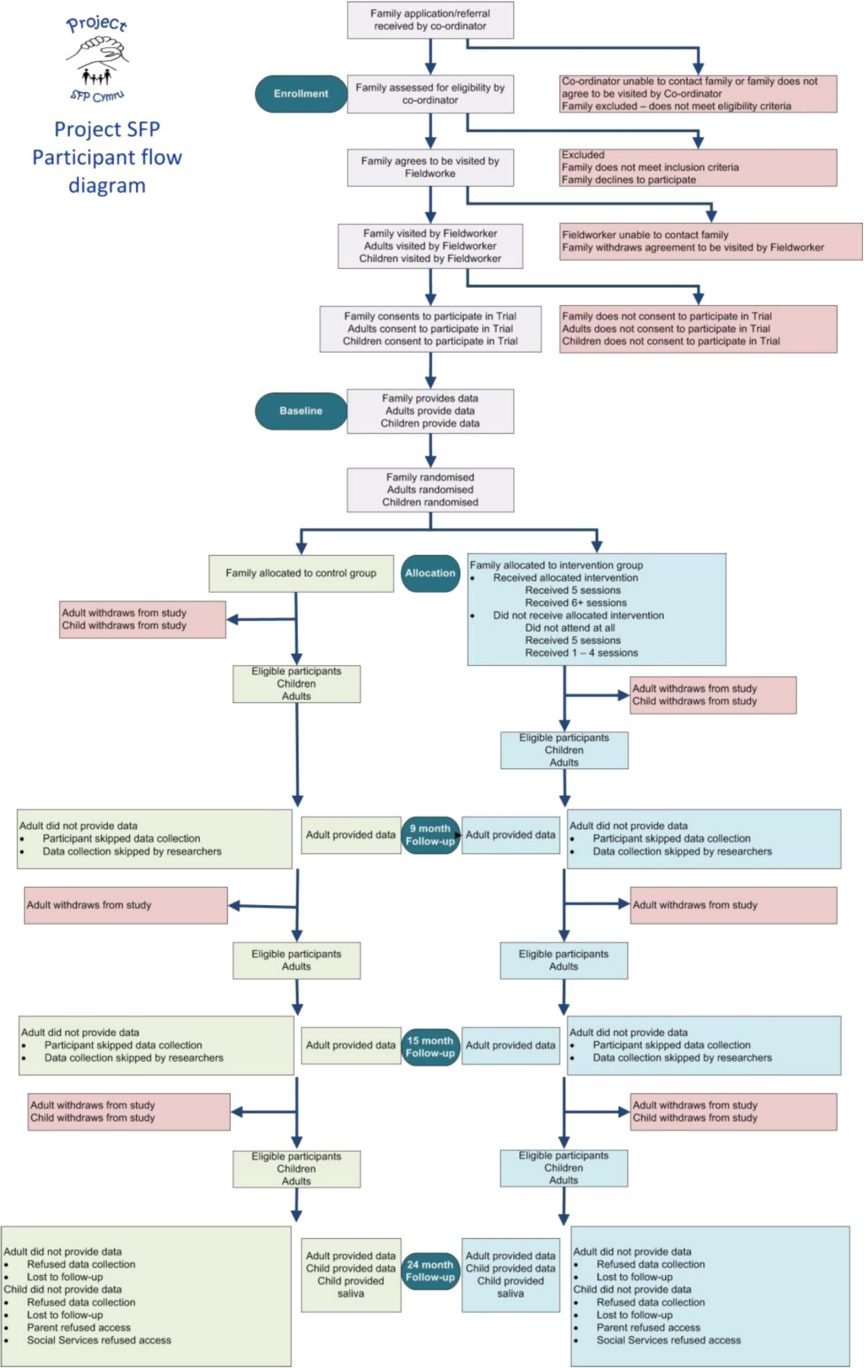
Participant flow diagram.

Potential referrers and families are informed that the Programme is being run as part of a trial and are provided with brief information about the trial. When a family is referred or applies to the SFP 10–14 they will be visited by a member of the programme delivery team (normally the programme coordinator) who will undertake a needs and eligibility assessment. Based on the information contained in the family referral/application form and the needs and eligibility assessment the coordinator determines if eligible families are from the ‘general population’ or a family with challenges (in the context of a group setting). If families are deemed eligible to attend the programme they will be asked if they are willing for a member of the research team to have access to their referral notes and to visit them. Where the family agrees a research fieldworker will then visit them to obtain informed consent to participate in the trial, collect baseline data using a computer assisted personal interview (CAPI), and inform the family of the treatment group (C or I) to which they have been randomised. Figure 1 shows the anticipated flow of participants through the trial.

We will offer all parents and young people who provide data at 2 year follow-up a £10 gift voucher. All adults who take part in the 9 and/or 15 month interviews will be entered in to a prize draw with the chance to win a £50 family outing. Adults have a 1 in 20 chance of winning.

### Participants

#### Inclusion and exclusion criteria

Table 1 describes the trial’s inclusion and exclusion criteria. Any families deemed eligible to attend SFP 10–14 UK will be included in the research trial, subject to their giving consent. Only families agreeing to participate in the trial and randomised to receive the intervention will have access to SFP 10–14 UK.

**Table 1:** Trial inclusion and exclusion criteria

**Inclusion criteria**	**Exclusion criteria**
At least one parent/carer and one child are willing to attend the programme together	Situations where either a parent or child does not want to attend the programme
The ability to speak English (help can be provided for parents or children with low literacy levels). Some programmes may also be delivered through the medium of Welsh if there is sufficient demand	Parents or children who cannot speak English (or Welsh, where appropriate)
A programme is being offered at a location to which it is practicable for a family to travel (as determined by the programme coordinator) within the next three months	No programme is being offered at a location to which it is practicable for a family to travel (as determined by the programme coordinator) within the next three months. In such a case the family would not be excluded. They will be placed on a waiting list for the programme and will be contacted when a programme is available. They will then be recruited into the trial
Families with a child aged 10-14	-
-	Families who do not live together - e.g. the child/children are in care
-	Families with very high needs or challenges (such as serious substance misuse problems, family breakdown or crisis)

### Ethical approval

Ethical approval for the trial was given by the Research Ethics Committee for Wales (reference 09/MRE09/53).

### Informed consent

Prior to being visited by a research fieldworker, all families who are invited to participate in the research trial will be mailed an information sheet. This will include specific documents for parents/carers and young people. There will also be information for parents/carers about our request to involve their children in the trial. The fieldworker will go through this information with families and address any queries or concerns that they may have.

Informed consent will be obtained from all participants. For a family to be included in the research trial at least one parent/carer must consent to participate, and parent/carer consent for the inclusion of at least one young person in the trial is required. Young people will also need to give consent for their participation in the research trial. The fieldworker will be careful not to offer, or in any way imply access to the programme.

### Randomisation

Families agreeing to participate in the research trial and who provide baseline data will be randomised using a computerised randomisation facility that will randomise families within strata defined by area (7) and use minimisation on the following variables:

• the category to which the family have been assigned (general population/family with challenges);

• age of children wishing to attend the programme (<12/12+). A mean will be taken if there is more than one child; and

• the number of children in the family wishing to attend the programme (1/>1).

There will be complete concealment of the randomisation sequence, set up securely within the randomisation database by the statistician, from the field recruitment staff and other trial team members. A random element, set at 80%, will also be used to increase the integrity and reduce the deterministic nature of the randomisation process.

### Intervention

Participating families will be randomised to one of two treatment groups. Either they will receive usual care with full access to existing services and a minimal information leaflet (control group-C), or they will receive the SFP 10–14 UK programme in addition to usual care with full access to existing services (intervention group – I). There will not be a defined programme of usual care, and the existing variation in services available to participants across the participating areas will continue throughout the trial period.

SFP 10–14 is open to families from a loosely defined and fairly large geographical area. The ‘mixed families’ approach aims to recruit about 4 families with challenges per group, and 6–8 families from the ‘general population’. Self-referrals (applications by families) come forward from awareness-raising in community and educational settings. Families are therefore drawn from the same area facilitating mutual support between sessions and at the end of the programme, but are not normally from a small or defined community such as a school or local neighbourhood. The programme comprises seven weekly sessions of two hours and is delivered in a range of community venues by a multi-agency team of trained professionals. In each session there is an hour during which parent sessions and young people sessions are conducted separately, followed by a second combined family hour. Typically the first hour focuses on skills (e.g. peer resistance for the young people, parenting for the parents), with the second hour designed to enable parents and young people to focus on communication skills, recognise family strengths, and practice skills covered in the first hour [27].

### Objectives

The trial’s primary objective is to assess the effectiveness of the SFP 10–14 UK in preventing alcohol misuse in young people. Its secondary objectives are to assess the programme’s impact on drug misuse, smoking behaviour, alcohol initiation and drink-related problems and school performance, among young people. Tertiary objectives are to measure the extent to which SFP 10–14 UK has effects on mental health and wellbeing, and protective factors for alcohol and tobacco use/misuse located in the family (e.g. family functioning, parenting and young people’s peer resistance skills). The trial will measure the costs associated with programme delivery and whether it is cost effective. It will assess if there are important variations in delivery and receipt across trial sites, and identify key programme theory, content and processes.

### Outcome measures

The trial has two **primary outcomes** - the number of occasions that young people report having drunk alcohol in the last 30 days; and drunkenness during the last 30 days, dichotomised as ‘never’ and ‘1-2 times or more’. **Secondary outcomes** (mainly concerned with long-term alcohol/tobacco/substance behaviours) are: reported use of cannabis (ever vs. never); weekly smoking (yes vs no, validated by salivary cotinine measures); age of alcohol use initiation; frequency of drinking more than 5 drinks in a row in the last 30 days; frequency of different types of alcoholic drinks; alcohol-related problems; and General Certificate of Secondary Education (GCSE) performance at age 15/16 (number of GCSEs passed and grades achieved, measured as a continuous outcome). All primary and secondary outcomes are collected from children at 2 year follow-up, with the exception of GCSE results, which we propose to collect via the Secure Anonymised Information Linkage (SAIL) Databank [60], once all participants have completed GCSEs/left compulsory education. The trial’s **tertiary outcomes** (mainly concerned with health and family wellbeing, and also substance use initiation) are: parenting (General Child Management Scale child report); family functioning (Family Relationship Index); children’s wellbeing and stress (Strengths and Difficulties Questionnaire scores); children’s health status (SF-36); parents/carers’ health status (GHQ and EQ5D); indicators of relative cost-effectiveness (derived from the health data); children’s smoking status (i.e. whether they have ever smoked/smoke now); young people’s self-efficacy; age of first cigarette; and age of first drug use. Tables 2, 3, 4, 5, and 6 provide a description of the trial’s primary, secondary and tertiary outcomes, and the specific measures used in each of the data collections. Selection of outcomes at 9, 15 and 24 months was informed by a literature review undertaken as part of the process evaluation.

**Table 2:** Measures collected at baseline from young people

**Domain/question topic**	**Purpose**	**Measure/source**
**Personal information**		
Gender, school year, age, DOB, place of birth, ethnicity and cohabitants	DE	Adapted from general household survey [61]
Family affluence	DE	Family affluence scale [62,63]
**Substance use**		
Ever smoked?	CV	One question from substance initiation index [29]
Smoking status	CV	ASSIST study version of NatCen/NFER question [64]
Ever drunk a drink?	CV	NatCen/NFER [65]
Age of first drink?	CV	NatCen/NFER [65]
Frequency of drinking	CV	NatCen/NFER [65]
Ever little drunk?	CV	Adapted from HBSC questionnaire
Little drunk in last 3 months?	CV	Adapted from HBSC questionnaire
Ever really drunk?	CV	Adapted from HBSC questionnaire
Really drunk in last 3 months?	CV	Adapted from HBSC questionnaire
Ever taken drugs	CV	Adapted from HBSC / NatCen/NFER
**Behaviour**		
Strengths and difficulties	CV	SDQ [66]
Aggressive and destructive conduct	CV	From Spoth, et al. [28]
**Life at home**		
Things in your bedroom	CV	Adapted from young people, new media survey [67,68]
TV, computer games, computer use	CV	From HBSC questionnaire
Free time activities	CV	Modified questions from west of Scotland twenty-07 study
Family functioning	CV	Family relationship index [69,70]
Parenting/child management	CV	General child management, project family [71,72]
Parents and school	CV	Three questions, one of which is adapted from the child rearing practices measure
Family activities	CV	From HBSC questionnaire/PEACH study
**General health**		
Sleep – difficulties	CV	From HBSC questionnaire
Getting up/bedtimes	CV	From PEACH study
Health state today	CV, HE	EQ-5D [73]
General health	CV	Kidscreen 27 [74]

**Key**: **CV** = Covariate; **HE** = Health Economics; **DE** = Demographics; **IO** = Immediate outcome; **ST** = Short term outcome; **PO** = Primary outcome; **SO** = Secondary outcome; **TO** = Tertiary outcome.

**Table 3:** Measures collected at baseline from parents/carers

**Domain/question topic**	**Purpose**	**Source**
**Personal information**		
Place of birth, relationship status, ethnicity, qualifications,	DE	Adapted from general household survey [61]
Employment	DE	NS-SEC
Co-habitants	DE	Adapted from GHS [61]
**Substance use**		
Smoke (Y/N)	CV	
Cigarettes smoked per day	CV	Heaviness of smoking index [75]
Frequency of drinking	CV	Adapted from AUDIT-C [76]
Number of drinks consumed when drinking	CV	Adapted from AUDIT-C [76]
Frequency of drinking 6+ drinks in a row	CV	Adapted from AUDIT-C [76]
Ever used drugs	CV	Adapted from HBSC/NatCen/NFER [65]
**Child’s substance use**		
Has child ever smoked?	CV	Question adapted from substance initiation index [29]
Does child smoke now?	CV	Adapted from NatCen/NFER [65]
Has child ever had a drink?	CV	Adapted from NatCen/NFER [65]
Has child ever been drunk?	CV	Developed by project SFP Cymru research team
Has child ever used drugs?	CV	Adapted from HBSC/NatCen/NFER [65]
**Strengths and difficulties**		
Strengths and difficulties	CV	SDQ [66]
**Section 6**		
Free time – use of TV, computer, etc.	CV	From HBSC questionnaire
Family activities	CV	From HBSC/11-16 West of Scotland adult questionnaire
Family functioning	CV	Family relationship index [69,70]
Parenting/child management	CV	General child management measure, project family [71,72,77]
Parent–child bonding	CV	Adapted from Spoth’s rural urban cumulative risk index and Arthur, et al. [78,79]
Parents and school	CV	Questions developed by project team and one from Conger’s child rearing practices measure
**Measures of health status**		
Health status	HE	GHQ-12 [80,81]
Health	HE	1 item from SF-36 [82]
Health state today	HE	EQ-5D [73]

**Table 4:** Measures collected at 9 and 15 month follow-up from parents/carers

**Domain/question topic**	**Purpose**	**Source**
**Life at home**		
Parenting/child management	IO	General child management measure, project family [71,72,77]
Parent–child bonding	ST	Adapted from Spoth’s rural urban cumulative risk index and Arthur, et al. [78,79]
Parents and school	ST	Questions developed by project team and one from Conger’s child rearing practices measure
Parental expectations	IO	Adapted from ALSPAC study/developed by project SFP Cymru research team
Participation in household tasks	IO	Adapted from ALSPAC/developed by project SFP Cymru research team
**Service utilisation**		
Health service utilisation	HE	Adapted from ALSPAC
Social care for adults	HE	Adapted from ALSPAC
Social care for children	HE	Adapted from ALSPAC
Criminal justice	HE	Adapted from ALSPAC

**Table 5:** Measures collected at 24 month follow-up from young people

**Domain/question topic**	**Purpose**	**Measure/source**
**Behaviour - strengths and difficulties**		
Strengths and difficulties/wellbeing and stress	TO	SDQ [66]
**Life at home**		
Family activities (opportunities for involvement in pro-social activities; possibly bonding)	ST	From HBSC/PEACH study
Young people’s own time		From west of Scotland Twenty 07
Parenting/child management	IO/	General child management measure, project family [71,72,77]
	TO	
Parents and school		Three questions; one adapted from the child rearing practices measure
Help around the home	IO	Adapted versions of questions asked to parents at 9/15 months.
Development of self-efficacy	ST/ TO	Bandura’s self efficacy scale [83]
Attachment to parents	ST	The security scale [84]
Befriending pro-social/anti-social peers	ST	Social development model scale on friends’ anti-social qualities (Interaction with antisocial peers scale) [85]
Positive bonding to school	ST	School bonding measure/SDM [86]
**Participant substance use**		
Smoking behaviour – ever smoked?		Question from substance initiation index [29]
Smoking status	TO/ SO	ASSIST study version of NatCen/NFER question [64]
Age first smoked	TO	From HBSC questionnaire
Ever drunk a drink?		NatCen/NFER [65]
Age of first drink	SO	Adapted from HBSC/NatCen/NFER
Drinking frequency, last month (including different types)	SO	Adapted questions from HBSC questionnaire
Ever really drunk?		From HBSC questionnaire
Age first drunk		Adapted version of question in HBSC questionnaire
Number of times drunk, drinking alcohol or 5+ drinks in a row in last month	PO/SO	Adapted from HBSC questionnaire
Alcohol-related problems	SO	Q21 from ESPAD survey [87]
Drug use – ever used?		Amended from HBSC/NatCen/NFER [65]
Cannabis use (ever, 12 months, or 30 days)	SO	From HBSC questionnaire
Age of first drug use	TO	Adapted from HBSC questionnaire
**General health**		
Health state today	HE/TO	EQ-5D (child version) [73]
General health	HE/TO	UK Kidscreen 10 [74]

**Table 6:** Measures collected at 24 month follow-up from parents/carers

**Domain/question topic**	**Purpose**	**Source**
**Section 2 – consent**		
**Life at home**		
Family activities		From HBSC/11-16 West of Scotland adult questionnaire
Family functioning	TO	Family relationship index [69,70]
Parenting/child management	IO	General child management measure, project family [71,72,77]
	SO	
Parent–child bonding	ST	Adapted from Spoth’s rural urban cumulative risk index and Arthur, et al. [78,79]
Parents and school		Question developed by project team and one from Conger’s child rearing practices measure
**Participant substance use**		
Smoking behaviour		Heaviness of smoking index [75]
Alcohol use		Adapted AUDIT-C questions, as used by Pre-empt study [76]
Drug use		Adapted from HBSC/NatCen/NFER [65]
**Health**		
Health status	HE	GHQ [80,81]
	TO	
Health	HE	1 item from SF-36 [82]
Health state today	HE	EQ-5D [73]
	TO	
**Service utilisation**		
Health, social care, education and criminal justice service utilisation	HE	Modified from ALSPAC

### Assessments and data collection

#### Baseline

Computer assisted personal interviews (CAPIs) are conducted by research fieldworkers with parents/carers and children. Children’s baseline CAPIs cover: substance use behaviours, and aggressive and destructive behaviour; strengths and difficulties (using the Strengths and Difficulties Questionnaire); health, and sleeping patterns. Five sets of questions assess aspects of participants’ home life, namely: possessions the participants have in their bedroom; family activities and free time activities; family functioning; and parenting/child management. Full details are provided in Table 2.

Parent/carer interviews cover: drug and tobacco use, and parental reports of children’s substance use; parent–child bonding; parenting and family relationships and activities: and parents/carers’ health status. Participants also complete the parent version of the Strengths and Difficulties Questionnaire (SDQ). Table 3 provides a full list of questions and measures included in the interviews.

### Follow-up

#### 9 and 15 months

Parents/carers are followed up at 9, and 15 months past baseline, using computer aided telephone interviews (CATIs) conducted by the Participant Resource Centre at Cardiff University. Interviews at 9 and 15 months (Table 4) are identical, and collect data on hypothesised short term and intermediate outcomes. Baseline measures of parent–child bonding and parenting/child management are repeated. Parental expectations of their child(ren) and opportunities for the latter to participate in household tasks are assessed. Health, criminal justice and social care services utilisation is captured.

#### 24 months

At two years past baseline CAPIs are conducted by research fieldworkers with parents/carers and children (Tables 5 and 6). Interviews with parents/carers cover participants’ substance use, health status and health and social care service utilisation. Young people’s interviews repeat baseline measures of smoking, alcohol and drug use. Additional questions examine age of first cigarette, age of first drunkenness, last month drinking frequency (including types of drinks), binge drinking and drunkenness frequency, cannabis use, and alcohol-related problems. The interview also assesses child reports of short term/intermediate outcomes, opportunities for involvement in pro-social activities in participants’ families, and attachment to parents, parenting/child management, and parental involvement in school work/activities. The extent to which young people help around the home is captured using suitably adapted versions of the questions asked to parents at 9/15 months. Other questions assess self-efficacy, school bonding, and interaction with anti-social peers. The child version of the SDQ is also administered. Measures of participants’ health used at baseline are repeated. Saliva samples are collected from consenting young people to assess inhalation of tobacco smoke in the previous few days, using a cotton wool swab of a salivette [88].

### Sample size

We will aim to recruit 378 families in each arm of the trial (756 families in total). This will provide us with at least 80% power at the 2.5% level (halved to account for our two primary outcomes) to detect either a 12% difference in young people reporting having drunk alcohol (assuming a control group prevalence of 48% [4]), or a 10% difference in young people reporting having been drunk (assuming a prevalence of 22% [4]), at two year follow-up. This sample size is based on an average family in the trial having 1.25 young people, an intracluster correlation coefficient (ICC) of 0.2 and is adjusted to allow for 25% loss to follow-up (including withdrawals from the trial by participants prior to 24 month follow-up).

### Analysis

#### Main analysis

The primary analyses will be based on the intention-to-treat principle and will involve fitting two-level logistic regression models to our primary outcomes (reported drinking at two year follow-up and reported drunkenness at two-year follow-up), with responses from young people nested within families. The covariates in the models include those balanced at randomisation (type of family – general population/family with challenges, and average age of young people) and baseline levels of drinking and drunkenness (depending on the outcome). Trial area (used as a stratification variable at randomisation) will be adjusted for. For each primary outcome, a statistically significant result will be concluded if the p-value for the trial arm explanatory variable is <0.025. While our power calculation is based on the above, a combined test will be performed if both p-values lie between 0.025 and 0.05. This will take the correlation of the outcomes into account and provide an overall p-value for the two outcomes.

The primary analyses will also be repeated

• controlling for any baseline imbalances;

• without controlling for any of the above mentioned covariates;

• respecting the original ordinal scale (the primary questions are asked on an ordinal scale ranging from 0 occasions to 40+ occasions) using an ordinal regression model; and

• using a complier average causal effect (CACE) approach, to investigate the treatment effect in the treatment adherent.

Secondary analysis includes investigating the effect of the intervention on substance use (reported use of tobacco and cannabis) at two year follow-up by fitting two-level logistic regression models, controlling for baseline reports of substance use, as well as the variables balanced at randomisation (all models will control for the variables that were balanced on at randomisation). The effect of the intervention on the proportion of young people reporting alcohol-related problems will be analysed similarly. The effect of the intervention on substance initiation (alcohol, tobacco and cannabis) will be investigated by fitting two-level Cox proportional hazards models (young people nested within families) for those participants who had not already initiated at baseline.

Educational achievement at 16 will be compared between trial arms by fitting a two-level logistic regression model (young people nested within families) comparing the proportion of young people who obtain five or more GCSEs at a grade C or above (GCSE Grades use an A*-G scale, with A* being the highest score). The proportion of young people who continue in education beyond 16 will be similarly compared between trial arms by fitting a logistic regression model. This analysis will be performed on the whole sample following the date that all participants (young people) should have completed school Year 11 when GCSEs are normally awarded. Other secondary and tertiary outcomes (e.g. parenting and child management, parent–child affective behaviour and wellbeing and stress) will be analysed by fitting similar regression models, controlling for clustering of responses within families (as appropriate) and controlling for variables that were balanced on at randomisation.

#### Sub-group & interim analysis

There is no planned interim analysis. Exploratory sub-group analyses are planned for:

• age of young person at baseline;

• gender of young person;

• smoking behaviour of young person at baseline;

• parental drinking behaviour at baseline;

• socio-economic status (using both the Family Affluence Scale and occupational status, which will be analysed separately);

• family status (general population/with challenges); and

• trial area.

Up to two subgroup analyses will be performed to test hypotheses generated from the process evaluation. These will be specified in a later version of the Statistical Analysis Plan (SAP) before any trial analysis takes place, and will be generated by researchers with no access to the trial outcome data. Subgroup analyses will also be performed to assess differential treatment effects for those who may be at high risk of substance initiation and misuse (based on baseline scores for SDQ, the Family Relationship Index and the General Child Management Scale).

### Process evaluation

An embedded process evaluation is examining how the programme is implemented and will facilitate interpretation of outcome effects [89]. In line with MRC’s guidelines [90], this component of the trial will enable the development and refinement of a programme logic model of the SFP10-14 UK by the research team, including its key processes, impacts and outcomes. The process evaluation has two key research questions: (1) How is the SFP 10–14 UK thought to influence social and individual behaviour of family members so that young people are less likely to use tobacco, misuse alcohol or to demonstrate other kinds of antisocial behaviour?; and (2) What are the best ways of implementing SFP 10–14 UK, and is there important variation in delivery and receipt? Following the framework proposed by Linnan and Steckler [91], the process evaluation will examine four key issues: trial implementation and context; trial arm fidelity; participation, reach and dose received/delivered; and reception and responsiveness. The process evaluation has the following aims, namely to:

• identify key programme content and processes;

• assess trial arm implementation and context;

• evaluate fidelity and completeness of programme delivery;

• assess participation and reach;

• calculate the extent of families’ attendance at SFP 10–14 UK sessions; and

• evaluate reception and response by families.

Multiple sources of evidence will be used to answer the research questions. Data collection will comprise:

• interviews with Welsh Government staff involved in programme commissioning (n = 1) and SFP training staff (n = 2), programme coordinators (n = 6), and coordinator managers (n = 6). Apart from coordinator managers, all these participants will be asked to take part in two interviews – once during the early period of programme delivery, and again towards the end;

• six focus groups (one in each delivery site) with staff involved in programme delivery (n = 48-60);

• completion of reflection sheets by facilitators after each session (n ≤ 336) to assess fidelity of delivery;

• researcher observation of programme sessions (three in each area, spread across the evaluation period). Two facilitators’ meetings (used to review and plan programme delivery) will be observed in each area; and

• 8–10 intervention group families will be invited to take part in focus groups in each area. Separate groups will be conducted with young people and parents/carers from the same families. Parents/carers from 8–10 control group families will also be invited to take part in a focus group in each area; and

• collection of routine data on programme recruitment, staffing and retention.

Table 7 shows how each of the data collection methods maps on to the aims and objectives.

**Table 7:** Process evaluation aims, objectives and methods

**Aim**	**Objectives**	**Literature review**	**Interview with Welsh government staff**	**Interviews with programme trainers**	**Interview with coordinators and managers**	**Focus groups with programme facilitators**	**Facilitator reflection sheets**	**Observation of programme sessions and facilitator meetings**	**Focus groups with intervention group families**	**Focus groups with control group parents**	**Routine data**	**Main trial data**
Inform decisions regarding which proximal outcomes should be captured at 9, 15 and 24 month follow-up interviews with parents/carers;	To develop a theoretical model of the SFP10-14 UK, specifying the social and behavioural hypotheses that underlie the programme.	**✓**										
	To use the theoretical model to predict proximal outcomes.											
Identify key programme content and processes;	To link proximal outcomes to components of implementation.	**✓**					**✓**	**✓**	**✓**			**✓**
	To compare scores for measures of hypothesised proximal and long-term outcomes from questionnaire respondents in intervention and control groups.											
	To revise and develop the logic model to take account of further hypotheses and priorities suggested by the data.											
	To determine how and when key aspects of delivery should be measured in order to assess fidelity to programme aims											
Assess trial arm implementation and context;	To describe implementation of the SFP10-14 UK, including characteristics of implementing agencies, staffing arrangements, referral routes and integration of services.			**✓**	**✓**	**✓**		**✓**	**✓**	**✓**	**✓**	**✓**
	To identify barriers and facilitators to implementation.											
	To identify family support services other than SFP10-14 UK used in trial areas.											
Evaluate fidelity and completeness of programme delivery;	For each area and programme run, to assess how closely implementation of SFP10-14 UK sessions matches the design and aims of SFP10-14 UK described in the programme manual			**✓**	**✓**	**✓**	**✓**	**✓**	**✓**	**✓**		
	To describe planned and actual roll out of SFP10-14 UK in each area.											
	To identify key factors influencing adherence											
	To estimate consistency in the provision of normal services											
Assess participation and reach;	In the intervention arm, to estimate the number of participants by different demographic groups, i.e. by gender; by age of children; by number of adults and children attending from each family; and by biological/other relationship of parents/carers to young people.										**✓**	**✓**
	In both trial arms, to estimate the number of study participants using family support services other than SFP10-14 UK, by demographic groups.											
Calculate the extent of families’ attendance at SFP10-14 UK sessions	To collate and summarise data showing (i) how many sessions are attended by each family; (ii) what proportion of the total number of enrolled family members attends each SFP10-14 UK session.				**✓**				**✓**		**✓**	
Evaluate reception and response by families	To explore parents/carers’ and young people’s experiences of attending the SFP and other services in terms of acceptability, their opinion of their value to them as individuals and any barriers or facilitators to participation.						**✓**	**✓**	**✓**	**✓**		

### Process evaluation analysis

Qualitative data from the process evaluation will be subjected to a thematic content analysis [92]. Key themes will be developed into an analytical framework. Each interview transcript will be entered into Atlas.ti 6, which will be used as a data management tool, permitting data coded to the same theme to be accessed quickly for further analysis. Each transcript will be coded to indicate the programme delivery location and type of participant, allowing analytical themes to be explored in relation to different groups’ experiences and to compare implementation across the seven areas and trial arms. Quantitative data on fidelity, participation and dose will be used in secondary analysis of outcome effects.

### Health economic evaluation

#### Aim

The aim of the economic evaluation is to assess the costs and effects associated with SFP 10–14 UK from the perspective of the UK Treasury - encompassing health and social services, education and criminal justice, and participating families, in a cost-consequences analysis. The cost-consequences analysis provides a comparative analysis of the alternative programmes available to participants (usual service provision only versus SFP 10–14 UK plus access to usual service provision) and a clear descriptive summary of the costs of SFP 10–14 UK and the consequences (outcomes for participants, service utilization, and utility gains). Effects will be reported separately enabling a full evaluation of the different outcome components (main trial outcomes, service utilisation, and utility gains) and related to the costs for both of the trial arms. Further, distinction will be made between the costs incurred in the delivery of SFP 10–14 UK and outcomes generated by it in each trial area (programme delivery site), so as to assess variation and potential for efficiency gains, if ‘lowest costs’ and ‘best outcomes’ were achieved across the board.

#### Methods

Costs will be categorised according to whether they are research or programme related, with discussions held with relevant staff to agree the attribution factors used to determine costs of SFP 10–14 UK. Further, the agencies that incur costs will be clearly specified, as will the agencies that benefit from reductions in resource utilisation, so as to enable inter-sectoral comparisons to be undertaken.

Based on service utilisation data collected from adult trial participants at 9, 15 and 24 month follow-up, all inputs and services provided by agencies will be documented in descriptive terms and wherever possible translated into monetary terms using appropriate published unit cost data (e.g. BNF [93], PSSRU [94], NHS Reference Costs [95]). A series of cost modules will be developed, to establish a profile of costs relating to the agency that incurred them. Overall costs of providing SFP 10–14 will be computed and will include programme set up costs, and costs of implementing and delivering the programme. The cost per participant and cost per family of attending SFP 10–14 UK will be computed for the trial and for each programme area. The costs of setting up SFP 10–14 UK will include promotional materials and resources involved in participant recruitment, in particular the number of hours spent promoting/raising awareness of the programme.

The costs of implementing and delivering SFP 10–14 UK will include staff time, venue and equipment costs, provision of support facilities, materials utilised etc. Participation in SFP 10–14 UK is likely to result in changes in the utilisation of other services provided by a range of agencies. These will be captured within the trial so as to identify the extent of costs offset as a result of the programme, and which represent one aspect of the outcomes generated by the programme. This will be done by collecting data on service utilisation from participants (and changes over time) at 9, 15 and 24 month follow-up. The combination of changes in service utilisation over time and costs of SFP 10–14 UK delivery will result in a net cost per participating family, which will be used in the assessment of the relative cost-effectiveness of the programme. The extent to which these changes in service utilisation are likely to be sustained over time will be modelled and quantified to produce scenarios for assessing the longer term efficiency of the programme.

Information regarding the number of participants requiring childcare support and transport to attend SFP 10–14 UK, and details of the job title and employer of programme staff who deliver each programme will be accessed from a monthly data return proforma submitted by each programme delivery team. Most data on the cost of delivering SFP 10–14 will be accessed via financial monitoring forms, submitted to the research team by the agencies with responsibility for leading programme delivery in each trial site. These forms capture all costs incurred by programme delivery teams, including those relating to specific SFP programmes (e.g. venue hire, refreshments, payment of participant travel expenses, provision of childcare for younger siblings taking part in the programme). As most programme facilitators are employed by a network of partner agencies (not the agency with primary responsibility for implementation), the associated staffing costs may not be available. If actual salary costs of facilitators cannot be obtained they will be derived from the hours worked documented on the Financial Monitoring Form and matched to the job title and employer information gathered via the Monthly Data Form. The salary associated with professional grade of each facilitator will be derived from relevant PSSRU unit cost data and multiplied by the hours worked to derive an estimate of staffing costs for facilitators. Where members of the research trial team organise or contribute to promotional events (e.g. information stands at school parents’ evenings) the costs incurred will be captured using a ‘Record of costs: promotional events' proforma. The provision of services other than SFP 10–14 UK to trial participants will be measured as part of 9, 15 and 24 month follow-up interviews with parents/carers. Table 8 provides a summary of cost categories and data sources and measures.

**Table 8:** Summary of cost categories and data sources and measures

**Cost category**	**Data source/measure**
**SFP implementation costs**	
Staff (including any recruitment/training costs)	Financial monitoring forms + PSSRU (2012)
Venue and equipment	Financial monitoring forms
Programme materials	Financial monitoring forms
Venue hire	Financial monitoring forms
Transport (for participants)	Financial monitoring forms
Refreshments	Financial monitoring forms
Childcare costs	Financial monitoring forms
Costs of any trips/pamper days arranged for families at the end of the programme	Financial monitoring forms
**Participant service resource utilisation (Unit costs)**	
GP surgery visit (Per patient contact lasting 11.7 minutes)	PSSRU (2012)
GP telephone consultation (Per telephone consultation lasting 7.1 minutes)	PSSRU (2012)
GP home visit (Per out of surgery visit lasting 23.4 minutes)	PSSRU (2012)
Community nurse - Home Visit (District nursing sister, District nurse)	PSSRU (2012)
Outpatient attendance	NHS reference Costs 2012
Inpatient attendance	NHS reference Costs 2012
Substance misuse services	Various + PSSRU (2012)
Mental health services	Various + PSSRU (2012)

#### Health economic analysis

##### Effects

The differences in primary, secondary and tertiary outcomes will represent the consequences of SFP 10–14 UK programme delivery to be used in conducting the cost consequences analysis. As part of this process the responses to EQ5D, collected from adults at baseline and 24 month follow-up, will be used to generate QALYs gained as a result of SFP 10–14 UK and compute the cost/QALY ratio.

##### Cost-consequences analysis

The cost consequences will be assessed once all necessary data has been collected, and post-trial modelling will be employed to assess the cost consequences over longer time horizons than is possible within the trial period. Changes in resources utilised over time will be calculated and used in conjunction with the costs of setting up and delivering SFP 10–14 UK to generate the overall cost of programme delivery per family, which will represent the incremental cost of providing the programme relative to usual service provision. The differences in primary, secondary and tertiary outcomes (including differences in utility scores derived from the EQ5D responses at each follow-up) will be used alongside the net cost of programme delivery in the cost consequences analysis to generate a set of indicators of relative cost-effectiveness within the trial period, based on incremental cost and incremental outcomes. Missing data will be dealt with by employing an appropriate imputation-based method for effectiveness and quality of life data [96], while the usual method for dealing with censored data relating to costs will be to employ the weighted cost method with known cost histories [97].

#### Sensitivity analysis

A series of one-way sensitivity analyses will be undertaken to assess the extent to which changes in the variables affect the baseline estimates. The variables will be adjusted in line with emerging distributions of data values, and impact on baseline estimates computed. A threshold analysis will also be undertaken to determine the degree of parameter variation required to alter conclusions derived from baseline findings. A series of scenarios will be developed and utilised within an economic model to assess the relative efficiency of SFP 10–14 UK over longer time horizons. Probabilistic sensitivity analysis will be undertaken to produce cost-effectiveness acceptability curves to assess the probability that SFP 10–14 UK represents value for money. Future costs and benefits will be discounted at the prevailing rate (currently 3.5% pa) to bring into present values. Costs and benefits will be discounted at 0% and 6% in the sensitivity analysis.

##### Modelling

The evaluation will also include a post-trial modelling phase, using a decision-analytic model, which will reflect longer time horizons than those available from within the trial period. The model will be populated with relevant information from within the trial period, but will also create a series of scenarios that reflect longer term costs and outcomes, based on parameter variation and discussion with experts.

### Public involvement

The MRC guidance on complex interventions recommends that “appropriate users should be involved at all stages of the development, process and outcome analysis of a complex intervention, as this is likely to result in better, more relevant science and a higher chance of producing implementable data” (p59) [90]. The involvement of the public has also been advocated in research policies to ensure that research is relevant, reliable and understandable [98,99]. Project SFP Cymru sits within the DECIPHer research centre which employs an Involving Young People Research Officer (IYPRO). This officer will be responsible for advising how public involvement is conducted throughout the trial.

All trial participants will be invited at baseline interview to be part of stakeholder groups. Parents and young people who consent will be contacted about the stakeholder events in their area. The frequency and content of the stakeholder events will vary dependent on the stage of the research and the need for lay input. It is envisioned that lay members will be consulted on trial materials such as questionnaires, the project website and newsletter, and identifying approaches to disseminating the findings of the trial to a lay audience, although flexibility will be built into the stakeholder events so that individuals can raise other issues they think are important for the research. The use of both top-down and bottom-up mechanisms of public involvement is designed to create a meaningful involvement process for all research participants. As recommended by INVOLVE, a national advisory organisation that aims to increase public involvement in NHS, public health and social care research, all lay members’ expenses will be covered, including travel and childcare costs, and vouchers will be given to compensate for time contributed [100]. We will also establish a stakeholders group to which all SFP coordinators will be invited, in order to promote effective communication between the research team and local practitioners.

## Discussion

Substance misuse is an important public health challenge and is associated with a range of short-term and long term harms. Positive family relationships and aspects of parenting (such as consistency, emotional warmth and monitoring) have been identified as key protective factors against young people’s misuse of alcohol, drugs and tobacco [101]. Prevention interventions which promote these protective factors therefore have the potential to make a significant impact on the health and wellbeing of young people. Such potential impacts cannot be assumed, and social interventions, like their clinical counterparts, may be ineffective in achieving their long term goals, or have unintended or sometimes harmful effects [54,102,103]. Prevention interventions therefore need to be theoretically grounded in their development, and subjected to rigorous evaluation before decisions about widespread implementation are made. This trial will assess the long-term effectiveness of the recently adapted UK version of the Strengthening Families Programme 10–14, including its impact on rates of alcohol consumption and drunkenness in young people aged 12–16 in a UK context. It seeks to understand how and why any identified outcomes occur, and to inform the future development of the programme in the UK.

Whilst parenting and family interventions are a key focus of UK social and public health policy, the majority of the interventions in this area have been developed in the United States [104-106]. It cannot be assumed that the effectiveness of an intervention in the country it is developed in will be replicated in new settings, or that programme logic models will operate in identical ways across different cultural contexts. Prevention interventions which are transported to new countries may require adaptation (which needs to balance fidelity with cultural relevance), and should be subject to rigorous evaluation to assess their effectiveness, and how key programme change processes function in new settings [107]. The results from this trial will help inform future decisions about the implementation of SFP 10-14 in the UK, and will demonstrate if the effectiveness of the programme in the US is replicated in a UK context using culturally adapted materials. It therefore contributes to parallel research endeavours on SFP 10-14 in a number of other countries [108-110].

As well as assessing the impact of the culturally adapted SFP 10-14 in the UK on long-term outcomes, the current trial is evaluating the implementation model developed in and being employed in Wales. A core aspect of this model is the strategy of retaining the SFP 10–14 as a universal prevention intervention (accessible to any family in a local community), and of forming programme groups to comprise a mix of families from the general population, and those who may experience/present challenges in a group setting. This model seeks to avoid some of the harmful effects of targeted interventions, including anti-social peer contagion [58]. Relatively few previous studies of parenting/family-based prevention programmes have examined strategies to enhance group dynamics, implementation fidelity or family engagement through group composition strategies [59]. Our process evaluation will allow us to understand whether the programme was delivered as intended (including the composition of SFP groups), what factors influenced this, and relationships between quality/adherence of programme delivery and outcomes (at a trial, area and family level). By embedding process and economic evaluations within the main trial we therefore aim to contribute to current knowledge about the value of universal prevention interventions, and the challenges of implementing family-based programmes.

In this trial we also aim to address some of the key methodological critiques of previous randomised controlled trials of the SFP10-14, particularly those concerning the use of multiple statistical tests, and the absence of a pre-specified primary outcome [41-43]. A key challenge when evaluating complex interventions such as SFP 10-14 is that they are designed to impact on multiple long-term health behaviours through enhancing manifold protective factors (parenting style, family communication processes, young people’s life skills), making the traditional trial design of a single primary outcome and small number of secondary outcomes inappropriate [90]. Thus we have specified two primary outcomes (related to drinking frequency and drunkenness frequency), alongside secondary and tertiary outcomes, and this is a strength of the trial design. The outcome measures map onto, and are designed to test a logic model for the programme developed by the research team, which has also driven the selection of short-term and intermediate outcomes. In this way the trial is assessing both the overall effectiveness of the SFP10-14, and its predicted causal pathways.

In order to maximise the external validity and utility of our findings we have adopted a pragmatic effectiveness trial design [111,112], in which recruitment procedures and delivery systems replicate ‘real world’ implementation. Thus in our trial of SFP 10–14 UK the programme is provided by local agencies that are likely to implement it in the future, and the research team does not seek to impose inclusion criteria with the aim of reducing the heterogeneity of the trial population (as might happen in an explanatory trial which attempts to recruit homogenous groups of participants). Participating families therefore comprise children from across the 10–14 age band which the programme is aimed at (12–16 at follow-up), and it is possible that the SFP10-14’s potential impact on health behaviours such as alcohol could be patterned according to age.

The trial team will need to obtain support from local agencies delivering SFP 10–14 for the trial, many of whom may have little or no previous experience of running parenting/family/substance misuse prevention programmes as part of an RCT. Practitioners (and others) may object to random allocation on the grounds that they believe that the intervention is likely to be effective [113], and that participants have unmet needs which SFP 10–14 UK addresses. Whilst central to many practitioners’ concerns may be the sense that participants randomised to the control condition are ‘given nothing’ (what Wong, et al. describe as ‘resentful demoralisation’ in the context of no treatment control groups) [114], in pragmatic trials such as ours, both intervention and control group families continue to receive ‘care as usual’ (i.e. whatever services or programmes are usually available), though usual care/services may differ across trial sites. One potential response of practitioners to allocation of participants to a control group is to offer a compensatory package of treatment or support [115], which can undermine the equivalence of the two arms of a trial. It will also be important for us to avoid contamination of the control group, and therefore ensure that families allocated to the control group are not offered SFP 10–14 UK prior to their 24 month follow-up interviews. We aim to build strong relationships with agencies involved in referring families to, and delivering SFP 10–14 through embedding research staff within these agencies, organising presentations to explain the rationale for the RCT design (and the need to maintain intervention and control groups and equivalence of access to usual care), and engaging the support of influential individuals (such as senior managers and team leaders). We have also sought to build strong partnerships with key policy makers, with the aim of generating high level support for the trial, and also increasing the likelihood that the results can be translated into policy and practice. In common with other comparable trials [116-118], we are likely to experience challenges with recruitment and retention of families. We plan to use a number of strategies to retain young people and parents/carers in the trial, including regular communication with participants, using agencies and schools to reach families who may have moved house after baseline data collection, forming groups of trial participants to advise us on the acceptability and likely impact of potential approaches, and offering incentives (e.g. vouchers, prize draws) at follow-up.

In conclusion, this trial aims to evaluate a promising family-based substance misuse prevention programme following its adaptation for the UK. The trial is designed to assess the effectiveness of SFP 10-14 UK, the extent to which it is delivered as intended (and key influences), and the costs and consequences of the programme. We aim to examine the relationship between trial outcomes and fidelity of implementation, and the value of the group composition strategies being used by SFP 10–14 UK practitioners in Wales.

## Competing interests

DF’s institution has received financial support for the development of the SFP 10–14 UK programme materials from the alcohol industry.

## Authors’ contributions

LM was the principal investigator (until 31^st^ October 2013). SM is the principal investigator from 1^st^ November 2013. LM, JSe, DF and SM conceived the study. LM, JSe, DF, DG, JH, KH, CP, IH, HRo, SM, ZR, and JSc contributed to the design of the study. JSe, LM, JH, DG, CP, IH, HRe, HRo and DF drafted the paper. All authors read and agreed the contents of the paper.

## Pre-publication history

The pre-publication history for this paper can be accessed here:

http://www.biomedcentral.com/1471-2458/14/49/prepub

## References

[B1] Colman I Murray J Abbott RA Maughan B Kuh D Croudace TJ Jones PB Outcomes of conduct problems in adolescence: 40 year follow-up of national cohort Br Med J 2009 338 a2981 10.1136/bmj.a2981 19131382 PMC2615547

[B2] Ezzati M Vander Hoorn S Lopez AD Danaei D Rodgers A Mathers CD Murray CJL Lopez AD, Mathers CD, Ezzati M, Jamison DT, Murray CJL Comparative quantification of mortality and burden of disease attributable to selected risk factors Global Burden of Disease and Risk Factors 2006 Washington DC: The World Bank / Oxford University Press 241 396 21250375

[B3] World Health Organisation Evidence-based strategies and interventions to reduce alcohol-related harm: global assessment of public-health problems caused by harmful use of alcohol 2007

[B4] Currie C Zanotti C Morgan A Currie D de Looze M Roberts C Samdal O Smith ORF Barnekow V Social determinants of Health and Well-Being among Young People. Health Behaviour in School-Aged Children (HBSC) Study: International Report from the 2009/2010 Survey Health Policy for Children and Adolescents 2012 Copenhagen: WHO Regional Office for Europe

[B5] Jackson CA Henderson M Frank JW Haw SJ An overview of prevention of multiple risk behaviour in adolescence and young adulthood J Public Health 2012 34 I31 I40 10.1093/pubmed/fdr113 22363029

[B6] NatCen Social Research and the National Foundation for Educational Research Smoking, drinking and drug use among young people in England in 2012 2013

[B7] Hale DR Viner RM Policy responses to multiple risk behaviours in adolescents J Public Health 2012 34 I11 I19 10.1093/pubmed/fdr112 22363026

[B8] Kipping RR Campbell RM MacArthur GJ Gunnell DJ Hickman M Multiple risk behaviour in adolescence J Public Health 2012 34 I1 I2 10.1093/pubmed/fdr122 22363025

[B9] Wild LG Flisher AJ Bhana A Lombard C Associations among adolescent risk behaviours and self-esteem in six domains J Child Psychol Psychiatr 2004 45 8 1454 1467 10.1111/j.1469-7610.2004.00330.x 15482505

[B10] Harakeh Z de Looze ME Schrijvers CTM van Dorsselaer SAFM Vollebergh WAM Individual and environmental predictors of health risk behaviours among Dutch adolescents: the HBSC study Public Health 2012 126 7 566 573 10.1016/j.puhe.2012.04.006 22607981

[B11] Catalano RF Hawkins JD Berglund ML Pollard JA Arthur MW Prevention science and positive youth development: competitive or cooperative frameworks? J Adolesc Health 2002 31 6 230 239 10.1016/S1054-139X(02)00496-2 12470920

[B12] Bond L Thomas L Toumbourou J Patton G Catalano R Improving the Lives of Young Victorians in Our Community: A Survey of Risk and Protective Factors 2000 Centre for Adolescent Health: Melbourne

[B13] Resnick MD Protective factors, resiliency and healthy youth development Adolesc Med 2000 11 1 157 165 10640344

[B14] Velleman R Alcohol Prevention Programmes: A Review of the Literature for the Joseph Rowntree Foundation (Part Two) 2009 Joseph Rowntree Foundation

[B15] Foxcroft DR Lowe G Adolescents' alcohol use and misuse: the socializing influence of perceived family life Drugs-Educ Prev Policy 1997 4 3 215 229

[B16] Garmiene A Žemaitiene N Zaborskis A Family time, parental behaviour model and the initiation of smoking and alcohol use by ten-year-old children: an epidemiological study in Kaunas, Lithuania BMC Public Health 2006 6 1 287 10.1186/1471-2458-6-287 17123446 PMC1665457

[B17] Moore GF Rothwell H Segrott J An exploratory study of the relationship between parental attitudes and behaviour and young people's consumption of alcohol Subst Abuse Treat Prev Policy 2010 5 6 10.1186/1747-597X-5-6 20412576 PMC2865449

[B18] Shortt AL Hutchinson DM Chapman R Toumbourou JW Family, school, peer and individual influences on early adolescent alcohol use: first-year impact of the resilient families programme Drug Alcohol Rev 2007 26 6 625 634 10.1080/09595230701613817 17943523

[B19] Velleman R Templeton LJ Substance misuse by children and young people: the role of the family and implications for intervention and prevention Paediatr Child Health 2007 17 1 25 30 10.1016/j.paed.2006.12.002

[B20] Cuijpers P Three decades of drug prevention research Drugs-Educ Prev Policy 2003 10 1 7 20 10.1080/0968763021000018900

[B21] Gillies V Meeting parents' needs? Discourses of 'support' and 'inclusion' in family policy Crit Soc Policy 2005 25 1 70 90 10.1177/0261018305048968

[B22] Edwards RT Ceilleachair A Bywater T Hughes DA Hutchings J Parenting programme for parents of children at risk of developing conduct disorder: cost effectiveness analysis Br Med J 2007 334 7595 682 685 10.1136/bmj.39126.699421.55 17350965 PMC1839236

[B23] Shulruf B O'Loughlin C Tolley H Parenting education and support policies and their consequences in selected OECD countries Child Youth Serv Rev 2009 31 5 526 532 10.1016/j.childyouth.2008.10.010

[B24] Welsh Assembly Government Working Together to Reduce Harm: The Substance Misuse Strategy for Wales 2008–2018 2008 Cardiff: Welsh Assembly Government

[B25] UK Home Office The Government's Alcohol Strategy 2012 London

[B26] Turner KMT Sanders MR Dissemination of evidence-based parenting and family support strategies: learning from the Triple P - Positive Parenting Program system approach Aggression and Violent Behavior 2006 11 2 176 193 10.1016/j.avb.2005.07.005

[B27] Molgaard VM Spoth R Redmond C Competency training: the strengthening families program for parents and youth 10–14 OJJDP Juvenile Justice Bulletin (NCJ 182208) 2000

[B28] Spoth RL Redmond C Shin C Reducing adolescents' aggressive and hostile behaviors - randomized trial effects of a brief family intervention 4 years past baseline Arch Pediatr Adolesc Med 2000 154 12 1248 1257 10.1001/archpedi.154.12.1248 11115311

[B29] Spoth RL Redmond C Trudeau L Shin C Longitudinal substance initiation outcomes for a universal preventive intervention combining family and school programs Psychol Addict Behav 2002 16 2 129 134 12079251

[B30] Spoth R Redmond C Shin C Azevedo K Brief family intervention effects on adolescent substance initiation: school-level growth curve analyses 6 years following baseline J Consult Clin Psychol 2004 72 3 535 542 15279537 10.1037/0022-006X.72.3.535

[B31] Spoth RL Shin C Redmond C Long-term effects of universal preventive interventions on methamphetamine use among adolescents Arch Pediatr Adolesc Med 2006 160 9 876 882 16953009 10.1001/archpedi.160.9.876

[B32] Spoth RL Trudeau LS Guyll M Shin C Benefits of universal intervention effects on a youth protective shield 10 years after baseline J Adolesc Health 2012 50 4 414 417 10.1016/j.jadohealth.2011.06.010 22443848 PMC3313466

[B33] Warner LA White HR Longitudinal effects of age at onset and first drinking situations on problem drinking Subst Use Misuse 2003 38 14 1983 2016 10.1081/JA-120025123 14677779

[B34] Zakrajsek JS Shope JT Longitudinal examination of underage drinking and subsequent drinking and risky driving J Safety Res 2006 37 5 443 451 10.1016/j.jsr.2006.06.002 17123546 PMC1853244

[B35] Jefferis BJMH Manor O Power C Social gradients in binge drinking and abstaining: trends in a cohort of British adults J Epidemiol Community Health 2007 61 2 150 153 10.1136/jech.2006.049304 17234875 PMC2465651

[B36] Spoth R Guyll M Day S Universal family-focused interventions in alcohol-use disorder prevention: cost-effectiveness and cost-benefit analyses of two interventions J Stud Alcohol 2002 63 2 219 228 12033699 10.15288/jsa.2002.63.219

[B37] Aos S Lieb R Mayfield J Miller M Pennucci A Benefits and Costs of Prevention and Early Intervention Programs for Youth 2004 Washington State: Washington State Institute for Public Policy

[B38] Caulkins JP Pacula RL Paddock SM Chiesa J School-Based Drug Prevention: What Kind of Drug Use Does it Prevent? 2002 Santa Monica, California: RAND Corporation

[B39] Foxcroft DR Ireland D Lister-Sharp DJ Lowe G Breen R Longer-term primary prevention for alcohol misuse in young people: a systematic review Addiction 2003 98 4 397 411 10.1046/j.1360-0443.2003.00355.x 12653810

[B40] Foxcroft D WHO Technical Report: Alcohol Misuse Prevention for Young People: a rapid review of recent evidence 2006 Oxford: Oxford Brookes University

[B41] Gorman DM Conde E Further comments on the path to drawing reasonable conclusions about prevention Addiction 2009 104 1 152 154 19133901 10.1111/j.1360-0443.2008.02383.x

[B42] Gorman DM Conde E Huber JC The creation of evidence in 'evidence-based' drug prevention: a critique of the strengthening families program plus life skills training evaluation Drug Alcohol Rev 2007 26 6 585 593 10.1080/09595230701613544 17943519

[B43] Midford R Is this the path to effective prevention? Addiction 2008 103 7 1169 1170 10.1111/j.1360-0443.2008.02224.x 18554349

[B44] Spoth R Trudeau L Redmond C Shin C Further clear examples of the need for more reasonable conclusions and critiques about prevention Addiction 2009 104 1 154 155

[B45] Spoth R Trudeau L Redmond C Shin C [Commentary] Finding a path to more reasonable conclusions about prevention: a response to Midford Addiction 2008 103 7 1171 1173 10.1111/j.1360-0443.2008.02270.x

[B46] White J Rumsey N Michie S Evidence of the Effectiveness of Interventions to Change Behaviours Related to Health in Young People Aged 11–18 2009 Department of Health: Commissioned by the Health Inequalities Unit

[B47] Allen D Coombes L Foxcroft DR Cultural accommodation of the strengthening families programme 10–14: UK Phase I study Health Educ Res 2007 22 4 547 560 17041022 10.1093/her/cyl122

[B48] Allen D Coombes L Foxcroft D Preventing alcohol and drug misuse in young people: adaptation and testing of the strengthening families programme 10–14 (SFP10-14) for use in the United Kingdom Oxford Brookes University: Oxford Undated

[B49] White C Warrener M Reeves A La Valle I Family intervention projects: an evaluation of their design, set-up and early outcomes 2008 London: National Centre for Social Research

[B50] Coombes L Allen D Marsh M Foxcroft D The strengthening families programme (SFP) 10–14 and substance misuse in Barnsley: the perspectives of facilitators and families Child Abuse Review 2009 18 1 41 59 10.1002/car.1055

[B51] Coombes L Allen D Marsh M Foxcroft D Implementation of the Strengthening Families (SFP) in Barnsley: The Perspectives of Facilitators and Families 2006 Oxford: Oxford Brookes University

[B52] Wiggins M Bonell C Burchett H Sawtell M Austerberry H Allen E Strange V Young People's Development Programme Evaluation: Final Report 2008 London: Social Science Research Unit, Institute of Education, University of London

[B53] Wiggins M Bonell C Sawtell M Austerberry H Burchett H Allen E Strange V Health outcomes of youth development programme in England: prospective matched comparison study Br Med J 2009 339 b2534 10.1136/bmj.b2534 19584408 PMC2714682

[B54] Gifford-Smith M Dodge KA Dishion TJ McCord J Peer influence in children and adolescents: crossing the bridge from developmental to intervention science J Abnorm Child Psychol 2005 33 3 255 265 10.1007/s10802-005-3563-7 15957555 PMC2747364

[B55] Gottfredson D Kumpfer K Polizzi-Fox D Wilson D Puryear V Beatty P Vilmenay M The strengthening Washington D.C. families project: a randomized effectiveness trial of family-based prevention Prev Sci 2006 7 1 57 74 10.1007/s11121-005-0017-y 16555144

[B56] Botvin GJ Griffin KW School-based programmes to prevent alcohol, tobacco and other drug use Int Rev Psychiatry 2007 19 6 607 615 10.1080/09540260701797753 18092239

[B57] Palinkas LA Atkins CJ Miller C Ferreira D Social skills training for drug prevention in high-risk female adolescents Prev Med 1996 25 6 692 701 10.1006/pmed.1996.0108 8936571

[B58] Dishion TJ Tipsord JM Peer contagion in child and adolescent social and emotional development Annu Rev Psychol, Vol 62 2011 62 189 214 10.1146/annurev.psych.093008.100412 PMC3523739 19575606

[B59] Segrott J Recruitment and group composition strategies for family-based substance misuse prevention interventions: an exploratory evaluation J Children's Services 2013 8 2 89 109 10.1108/JCS-03-2013-0007

[B60] Lyons RA Jones KH John G Brooks CJ Verplancke JP Ford DV Brown G Leake K The SAIL databank: linking multiple health and social care datasets BMC Med Inform Decis Mak 2009 9 3 10.1186/1472-6947-9-3 19149883 PMC2648953

[B61] Office for National Statistics General Household 2007 Survey Report - Appendix E Household and Individual Questionnaires 2009

[B62] Currie C Molcho M Boyce W Holstein B Torsheim T Richter M Researching health inequalities in adolescents: the development of the Health Behaviour in School-Aged Children (HBSC) Family Affluence Scale Soc Sci Med 2008 66 6 1429 1436 10.1016/j.socscimed.2007.11.024 18179852

[B63] Boyce W Torsheim T Currie C Zambon A The family affluence scale as a measure of national wealth: validation of an adolescent self-report measure Soc Indicators Res 2006 78 3 473 487 10.1007/s11205-005-1607-6

[B64] Campbell R Starkey F Holliday J Audrey S Bloor M Parry-Langdon N Hughes R Moore L An informal school-based peer-led intervention for smoking prevention in adolescence (ASSIST): a cluster randomised trial Lancet 2008 371 9624 10.1016/S0140-6736(08)60692-3 PMC2387195 18468543

[B65] National Centre for Social Research, National Foundation for Educational Research Smoking, Drinking and Drug Use Among Young People in England in 2008 Full Report 2009 NHS Information Centre for Health and Social Care

[B66] Goodman R The strengths and difficulties questionnaire: a research note J Child Psychol Psychiatry 1997 38 5 10.1111/j.1469-7610.1997.tb01545.x 9255702

[B67] Livingstone S Brober M UK children go online: surveying the experiences of young people and their parents 2004 London: LSE Research Online

[B68] Livingstone S Strategies of parental regulation in the media-rich home Comput Hum Behav 2007 23 2

[B69] Holahan CJ Moos RH Social support and adjustment - predictive benefits of social climate indexes Am J Community Psychol 1982 10 4 403 415 10.1007/BF00893979 7137128

[B70] Billings AG Moos RH The role of coping responses and social resources in attenuating the stress of life events J Behav Med 1981 4 2 10.1007/BF00844267 7321033

[B71] McMahon R Metzler CW Ashery RS, Robertson EB, Kumpfer KL Selecting parenting measures for assessing family based prevention interventions Drug Abuse Prevention through Family Interventions 1998 Rockville, MD: National Institute on Drug Abuse 294 323

[B72] Spoth R Redmond C Haggerty K Ward T A controlled parenting skills outcome study examining individual difference and attendance effects J Marriage Fam 1995 57 2 449 464 10.2307/353698

[B73] Rabin R de Charro F EQ-5D: a measure of health status from the EuroQol group Ann Med 2001 33 5 10.3109/07853890109002087 11491192

[B74] The Kidscreen Group Europe The KIDSCREEN Questionnaires: Quality of life questionnaires for children and adolescents: Handbook 2006 Lengerich: Pabst Science Publishers

[B75] Borland R Yong HH O'Connor RJ Hyland A Thompson ME The reliability and predictive validity of the heaviness of smoking index and its two components: findings from the international tobacco control four country study Nicotine Tob Res 2010 12 s1 s45 s50 20889480 10.1093/ntr/ntq038 PMC3307335

[B76] Bush K Kivlahan DR McDonell MB Fihn SD Bradley KA Ambulatory Care Quality Improvement P The AUDIT alcohol consumption questions (AUDIT-C) - an effective brief screening test for problem drinking Arch Intern Med 1998 158 16 1789 1795 10.1001/archinte.158.16.1789 9738608

[B77] Spoth R Redmond C Shin C Direct and indirect latent-variable parenting outcomes of two universal family-focused preventive interventions. Extending a public health-oriented research base Journal of Consulting Child Psychology 1998 66 2 385 399 10.1037//0022-006x.66.2.385 9583342

[B78] Spoth R Goldberg C Neppl T Trudeau L Ramisetty-Mikler S Rural–urban differences in the distribution of parent-reported risk factors for substance use among young adolescents J Subst Abuse 2001 13 4 609 623 10.1016/S0899-3289(01)00091-8 11775086

[B79] Arthur MW Hawkins JD Catalano RF Pollard JA Item-construct dictionary for the student survey of risk and protective factors and prevalence of alcohol, tobacco, and other drug use. Unpublished Technical Document 1995 Seattle, WA: University of Washington

[B80] Goldberg DP The Detection of Psychiatric Illness by Questionnaire 1972 London: Oxford University Press

[B81] Vieweg BW Hedlund JL The General Health Questionnaire (GHQ): a comprehensive review J Oper Psychiatry 1983 14 2 74 81

[B82] Ware JE Sherbourne CD The MOS 36-item short-form health survey (SF-36).1. Conceptual-framework and item selection Med Care 1992 30 6 473 483 10.1097/00005650-199206000-00002 1593914

[B83] Bandura A Pajares F, Urdan T, Greenwich CT A Guide for constructing self-efficacy scales Self-Efficacy Beliefs of Adolescents 2006 Information Age Publishing 307 337

[B84] Kerns KA Aspelmeier JE Gentzler AL Grabill CM Parent-child attachment and monitoring in middle childhood J Fam Psychol 2001 15 1 69 81 11322086 10.1037//0893-3200.15.1.69

[B85] Arthur M Hawkins J Catalano R Pollard J Student Survey of Risk and Protective Factors and Prevalence of Alcohol, Tobacco, & Other Drug Use Social Development Research Group, University of Washington

[B86] Hawkins JD Guo J Hill KG Battin-Pearson S Abbott RD Long-term effects of the Seattle social development intervention on school bonding trajectories Appl Dev Sci 2001 5 4 225 236 10.1207/S1532480XADS0504_04 17955057 PMC2040120

[B87] Hibell B Guttormsson U Ahlström S Balakireva O Bjarnason T Kokkevi A Kraus L The 2011 ESPAD Report: Substance Use Among Students in 36 European Countries 2011 Stockholm: The Swedish Council for Information on Alcohol and other Drugs (CAN)

[B88] Holliday JC Moore GF Moore LAR Changes in child exposure to secondhand smoke after implementation of smoke-free legislation in Wales: a repeated cross-sectional study BMC Public Health 2009 9 430 10.1186/1471-2458-9-430 19930678 PMC2789068

[B89] Springett J Appropriate approaches to the evaluation of health promotion Crit Public Health 2001 11 2 139 151 10.1080/09581590110039856

[B90] Craig P Dieppe P Macintyre S Michie S Nazareth I Petticrew M Developing and Evaluating Complex Interventions: New Guidance 2008 Medical Research Council

[B91] Linnan L Steckler A Steckler A, Linnan L Process evaluations for public health interventions and research Process Evaluation for Public Health Interventions and Research 2002 San Francisco, CA: Jossey-Bass

[B92] Braun V Clarke V Using thematic analysis in psychology Qual Res Psychol 2006 32 2 77 101

[B93] British national formulary [http://www.bnf.org/bnf/index.htm]

[B94] PSSRU - personal social services research unit [http://www.pssru.ac.uk/index-kent-lse.php]

[B95] Department of Health Reference costs guidance for 2012–13 2013

[B96] Brazier J Ratcliffe J Salomon JA Tsuchiy A Measuring and Valuing Health Benefits for Economic Evaluation 2007 Oxford: Oxford University Press

[B97] Young TA Estimating mean total costs in the presence of censoring - A comparative assessment of methods Pharmacoeconomics 2005 23 12 1229 1242 10.2165/00019053-200523120-00007 16336017

[B98] Department of Health Research Governance Framework for Health and Social Care 2005 London: Department of Health

[B99] Department of Health Best Research for Best Health. A New National Health Research Strategy 2006 London: Department of Health

[B100] INVOLVE What you need to know about payment: an introductory guide for members of the public who are considering active involvement in NHS, public health or social care research 2011 Eastleigh: INVOLVE

[B101] Sandler IN Schoenfelder EN Wolchik SA MacKinnon DP Long-term impact of prevention programs to promote effective parenting: lasting effects but uncertain processes Annu Rev Psychol, Vol 62 2011 62 299 329 10.1146/annurev.psych.121208.131619 PMC3061231 20822438

[B102] McCord J Cures that harm: unanticipated outcomes of crime prevention programs Ann Am Acad Pol Soc Sci 2003 587 16 30 10.1177/0002716202250781

[B103] Petrosino A Turpin-Petrosino C Finckenauer JO Well-meaning programs can have harmful effects! Lessons from experiments of programs such as scared straight Crime & Delinquency 2000 46 3 354 379 10.1177/0011128700046003006

[B104] Petrie J Bunn F Byrne G Parenting programmes for preventing tobacco, alcohol or drugs misuse in children <18: a systematic review Health Educ Res 2007 22 2 177 191 16857779 10.1093/her/cyl061

[B105] Woolfenden SR Williams K Peat JK Family and parenting interventions for conduct disorder and delinquency: a meta-analysis of randomised controlled trials Arch Dis Child 2002 86 4 251 256 10.1136/adc.86.4.251 11919097 PMC1719168

[B106] Dretzke J Frew E Davenport C Barlow J Stewart-Brown S Sandercock J Bayliss S Raftery J Hyde C Taylor R The effectiveness and cost-effectiveness of parent training/education programmes for the treatment of conduct disorder, including oppositional defiant disorder, in children Health Technol Assess 2005 9 50 10.3310/hta9500 16336845

[B107] Castro FG Barrera M Martinez CR The cultural adaptation of prevention interventions: resolving tensions between fidelity and fit Prev Sci 2004 5 1 41 45 15058911 10.1023/b:prev.0000013980.12412.cd

[B108] Stolle M Stappenbeck J Wendell A Thomasius R Family-based prevention against substance abuse and behavioral problems: culture-sensitive adaptation process for the adaptation of the US-American Strengthening Families Program 10–14 to German conditions Am J Public Health 2011 19 4 389 395 10.1007/s10389-011-0405-7

[B109] Okulicz-Kozaryn K Foxcroft DR Effectiveness of the strengthening families programme 10–14 in Poland for the prevention of alcohol and drug misuse: protocol for a randomized controlled trial BMC Public Health 2012 12 319 10.1186/1471-2458-12-319 22551472 PMC3379928

[B110] Skärstrand E Larsson J Andréasson S Cultural adaptation of the strengthening families programme to a Swedish setting Health Educ 2008 108 4 287 300 10.1108/09654280810884179

[B111] Roland M Torgerson D Understanding controlled trials: what are pragmatic trials? Br Med J 1998 316 285 10.1136/bmj.316.7127.285 9472515 PMC2665488

[B112] Murphy SM Edwards R Williams N Raisanen L Moore G Linck P Hounsome N Din N Moore L An evaluation of the effectiveness and cost effectiveness of the National Exercise Referral Scheme in Wales, UK: a randomised controlled trial of a public health policy initiative. (vol 66, pg 745,2012) J Epidemiol Community Health 2012 2012 66(11) 10.1136/jech-2011-200689 PMC3402741 22577180

[B113] Macintyre S Good intentions and received wisdom are not good enough: the need for controlled trials in public health J Epidemiol Community Health 2011 65 7 564 567 10.1136/jech.2010.124198 21148137

[B114] Wong ICK Team R Randomised controlled trials (RCTs) to evaluate complex healthcare interventions - a case study Pharm World Sci 2004 26 5 247 252 15598063 10.1023/b:phar.0000042920.34663.04

[B115] Avins AL Cherkin DC Sherman KJ Goldberg H Pressman A Should we reconsider the routine use of placebo controls in clinical research? Trials 2012 13 10.1186/1745-6215-13-44 PMC3404895 22540350

[B116] Simkiss DE Snooks HA Stallard N Davies S Thomas MA Anthony B Winstanley S Wilson L Stewart-Brown S Measuring the impact and costs of a universal group based parenting programme: protocol and implementation of a trial BMC Public Health 2010 10 364 10.1186/1471-2458-10-364 20573236 PMC2905332

[B117] Prinz RJ Smith EP Dumas JE Laughlin JE White DW Barron R Recruitment and retention of participants in prevention trials involving family-based interventions Am J Prev Med 2001 20 1 31 37 10.1016/S0749-3797(00)00271-3 11146258

[B118] Heinrichs N Bertram H Kuschel A Hahlweg K Parent recruitment and retention in a universal prevention program for child behavior and emotional problems: barriers to research and program participation Prev Sci 2005 6 4 275 286 10.1007/s11121-005-0006-1 16075192

